# Comparative outcomes of pure laparoscopic and open donor right hepatectomy: the first report from a Southeast Asian transplant center

**DOI:** 10.1186/s12893-022-01507-0

**Published:** 2022-02-11

**Authors:** Worakitti Lapisatepun, Sunhawit Junrungsee, Anon Chotirosniramit, Kanya Udomsin, Warangkana Lapisatepun, Phuriphong Chanthima, Settapong Boonsri, Suraphong Lorsomradee

**Affiliations:** 1 Division of Hepatobiliary Pancreatic Surgery, Department of Surgery, Faculty of Medicine, Chiang Mai University, Chiang Mai, Thailand; 2 Excellence Center of Organ Transplantation, Faculty of Medicine, Chiang Mai University, Thailand, 110 Intavarorod, Sripoom, 50200 Chiang Mai, Thailand; 3 Department of Anesthesiology, Faculty of Medicine, Chiang Mai University, Chiang Mai, Thailand

**Keywords:** Living donor liver transplantation, Pure laparoscopic donor right hepatectomy, Conventional open donor right hepatectomy, Perioperative outcome, Indocyanine green

## Abstract

**Background:**

Pure laparoscopic donor right hepatectomy (PLDRH) can provide better operative outcomes for the donor than conventional open donor right hepatectomy (CODRH). However, the complexity of the procedure typically makes transplant teams reluctant to perform it, especially in low-volume transplant centers. We compared the outcomes of PLDRH and CODRH to demonstrate the feasibility of PLDRH in a low-volume transplant program.

**Methods:**

We carried out a retrospective study of adult living donor liver transplantation in Chiang Mai University Hospital from January 2015 to March 2021. The patients were divided into a PLDRH group and a CODRH group. Baseline characteristics, operative parameters, and postoperative complications of donors and recipients were compared between the two groups.

**Results:**

Thirty patients underwent donor hepatectomy between the dates selected (9 PLDRH patients and 21 CODRH patients). The baseline characteristics of the 2 groups were not significantly different. The median graft volume of the PLDRH group was 693.8 mL, which was not significantly different from that of the CODRH group (726.5 mL) The PLDRH group had a longer operative time than the CODRH group, but the difference was not statistically significant (487.5 min vs 425.0 min, p = 0.197). The overall complication rate was not significantly different between the two groups (33.3% vs 22.2%, p = 0.555). Additionally, for the recipients, the incidence of major complications was not significantly different between the groups (71.3 vs 55.6%, p = 0.792).

**Conclusion:**

Even in the context of this low-volume transplant program, whose staff have a high level of experience in minimally invasive hepatobiliary surgery, PLDRH showed similar results to CODRH in terms of perioperative outcomes for donors and recipients.

**Supplementary Information:**

The online version contains supplementary material available at 10.1186/s12893-022-01507-0.

## Background

Laparoscopic liver resection has become increasingly popular worldwide. The existing literature shows several significant advantages of laparoscopic liver resection over open liver resection (OLR), such as minimized blood loss and shorter hospital stays for patients [1]. Regarding liver transplantation, living donor liver transplantation (LDLT) is also a majority of liver transplant activity in Asian countries, particularly in geographical areas where deceased liver donor numbers are low, including the northern part of Thailand.

The first pure laparoscopic donor hepatectomy was left lateral sectionectomy for pediatric liver transplantation, reported by Cherqui et al. [2] Following this, many publications have demonstrated the benefit, safety and feasibility of laparoscopic donor left lateral sectionectomy (LLS) [3]. More recently, a panel of experts on laparoscopic liver surgery and LDLT stated that laparoscopic donor LLS should be the standard approach for pediatric liver transplantation. For adult LDLT, the right lobe of the liver is usually used with regard to body weight and severity of the recipient. Many studies have shown that pure laparoscopic donor right hepatectomy (PLDRH) is a feasible option that reduces postoperative complications and provides better early surgical outcomes than conventional open donor right hepatectomy (CODRH) [4, 5]. However, regarding the complexity of PLDRH, experts agree that the performance of this operation should be restricted to centers that possess expertise in complex hepatobiliary surgery, donor hepatectomy and advanced laparoscopic hepatobiliary surgery [6].

Chiang Mai University Hospital contains a high-volume hepatobiliary and pancreatic surgery center that has been serving the northern part of Thailand since 2005 and began its LDLT program and a minimally invasive liver resection program in early 2015. Right hepatectomy was performed on the donors using the conventional open technique with an intraoperative conventional cholangiogram until the end of 2019, when one of the transplant surgeons received training from a high-volume laparoscopic donor hepatectomy center (Seoul National University Hospital). Since early 2020, the department has used minimally invasive liver resection, including liver mobilization, parenchymal transection and hilar structure dissection; has started using real-time indocyanine green (ICG) cholangiograms to facilitate bile duct division instead of using the more conventional cholangiogram; and has started the first case of pure laparoscopic donor right hepatectomy in March 2020. To date, 9 cases of PLDRH have been performed in our center.

The aim of this study is to present the institutional experience of developing a safe and efficient pure laparoscopic donor right hepatectomy process for adult living donor liver transplantation without transition from hand- or laparoscopic-assisted donor hepatectomy. To the best of our knowledge, this study is the first report of PLDRH from a Southeast Asian transplant center.

## Materials and methods

This study was approved by the Research Ethics Committee of the Faculty of Medicine, Chiang Mai University (approval number 214/2564). Data were collected from all living liver donors who underwent donor right hepatectomy in the department between 1 January 2015 and 31 March 2021. Twenty-one donors underwent CODRH, and 9 donors underwent PLDRH by a single surgeon. There was no hand-assisted or laparoscopic-assisted donor right hepatectomy during this time period. There was also no ABO-incompatible liver transplant. Postoperative complications were classified and reported according to the Clavien–Dindo classification. [7]

### Donor evaluation and selection

The criteria for donors were an age of 18–60 years and an underlying medical conditions that are contraindications for surgery and liver donation.

Liver volume and vascular anatomy including the hepatic artery, portal vein and hepatic vein were evaluated using multiphase contrast-enhanced computed tomography. The future liver remnant was calculated by liver volumetry software (Synapse3D, Fuji company, Japan) and must exceed 25% of the total liver volume and be sufficient for the recipient by keeping the graft-to-recipient weight ratio (GRWR) more than 0.8. Biliary anatomy and degree of fatty liver were evaluated by magnetic resonance cholangiopancreatography (MRCP).

For the first few cases of CODRH, only donors who had no variations in their liver vasculature or biliary tract were selected; however, these limitations were abandoned after the experience of the team developed. The first 8 liver donors who underwent PLDRH had single right portal veins longer than 1 cm, a single right hepatic artery, a single right bile duct longer than 0.5 cm, and less than 2 inferior hepatic veins. There was no graft size limitation for PLDRH. However, the last case of PLDRH had a right posterior portal vein graft from the main portal vein.

### Pure laparoscopic donor right hepatectomy technique

**Fig. 1:**
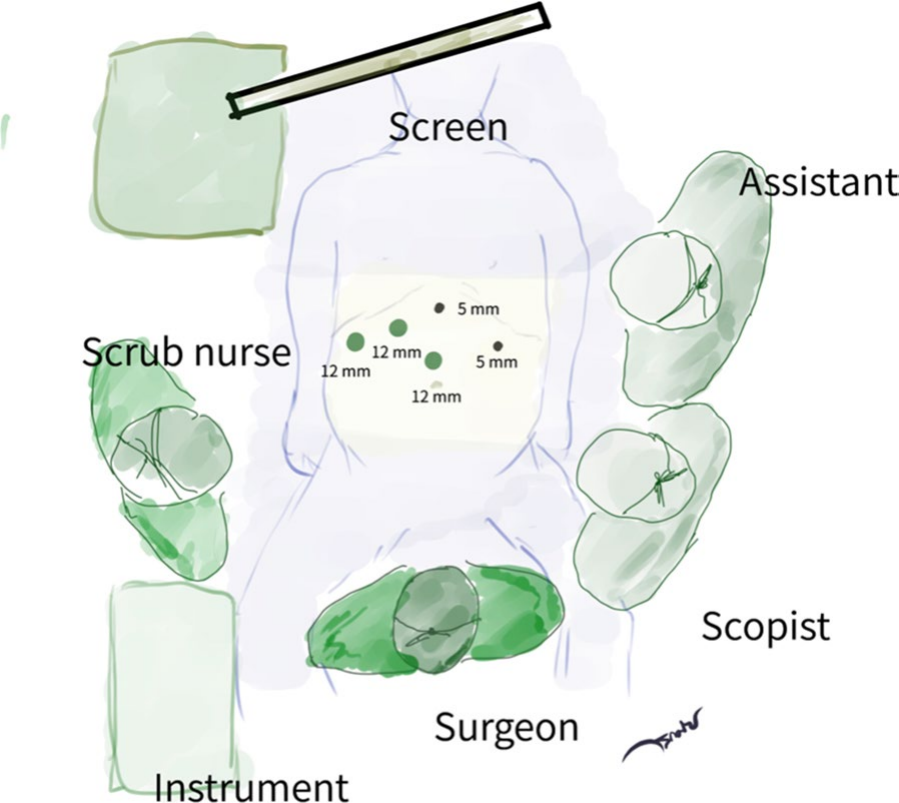
Patient, surgical team and port positions

The donor was placed supine in a reverse Trendelenburg position with their legs apart. The surgeon, assistant and scope operator were positioned as demonstrated in Fig. 1. A 12 mm. A camera port was inserted into the supraumbilical area using the open technique, and the pneumoperitoneum was gradually created and settled at 12 mmHg. A 30-degree scope with ICG light was used in all PLDRH cases. Two assistance ports were inserted under direct supervision, and the round ligament and falciform ligament were divided. Subsequently, two surgeon working ports were inserted to facilitate a precise parenchymal transection plane (Fig. 1).

The liver was completely mobilized, except for Makuuchi’s ligament. The anterior and lateral planes between the liver and inferior vena cava (IVC) were dissected, and the short hepatic veins were ligated. If there was an inferior hepatic vein, it was left in place to prevent liver congestion and ligated at the last step. No hanging maneuver was performed.

The next step was the hilar dissection. To acquire the best exposure of the hilar structure, the gallbladder and round ligament were retracted upward and outward by the assistant. Hilar dissection started with dissection of Calot’s triangle to safely double-ligate the cystic duct and ligate the cystic artery. The gallbladder was left in place to provide traction and exposure.

**Fig. 2:**
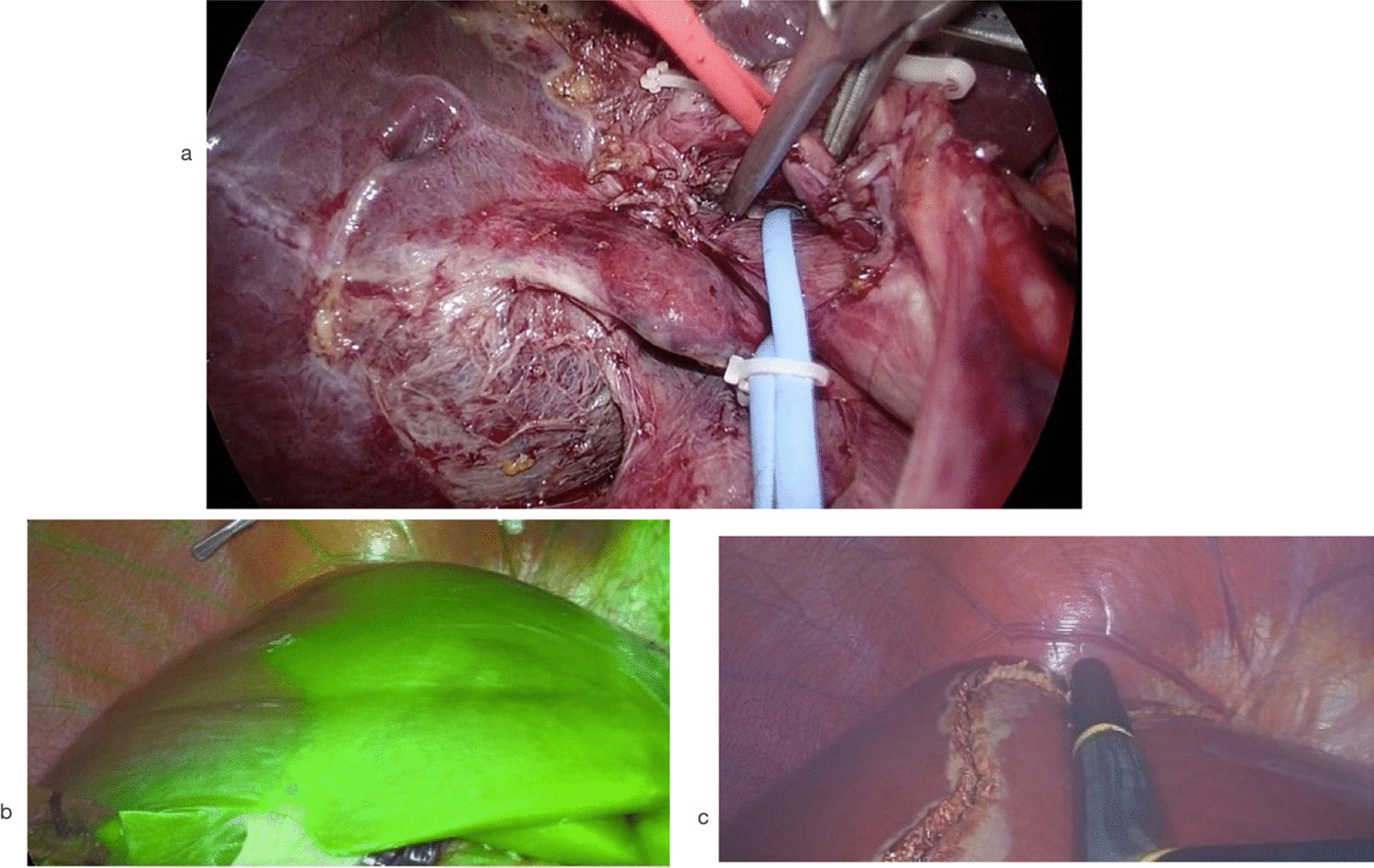
Vascular inflow identification and ischemic demarcation. **a** The right hepatic artery and right portal vein were wrapped with red and blue elastic tape, respectively, and then temporarily controlled with an endo-bulldog clamp. **b** Ischemic demarcation line demonstrated and marked by overlaid near-infrared imaging. **c** Laparoscopic ultrasound was used to identify the middle hepatic vein

The right hepatic artery (RHA) was dissected laterally to the common hepatic duct (CHD) and wrapped with elastic tape. The right portal vein (RPV) was then dissected until the portal vein bifurcation was clearly identified. The RPV was then wrapped with elastic tape, and the gallbladder was completely removed. After that, the RHA and RPV were temporarily clamped with an endo-bulldog clamp, and 2.5 mg of indocyanine green was injected intravenously. The transection line was clearly visualized and marked, and then the endo-bulldog clamps were removed (Fig. 2).

**Fig. 3:**
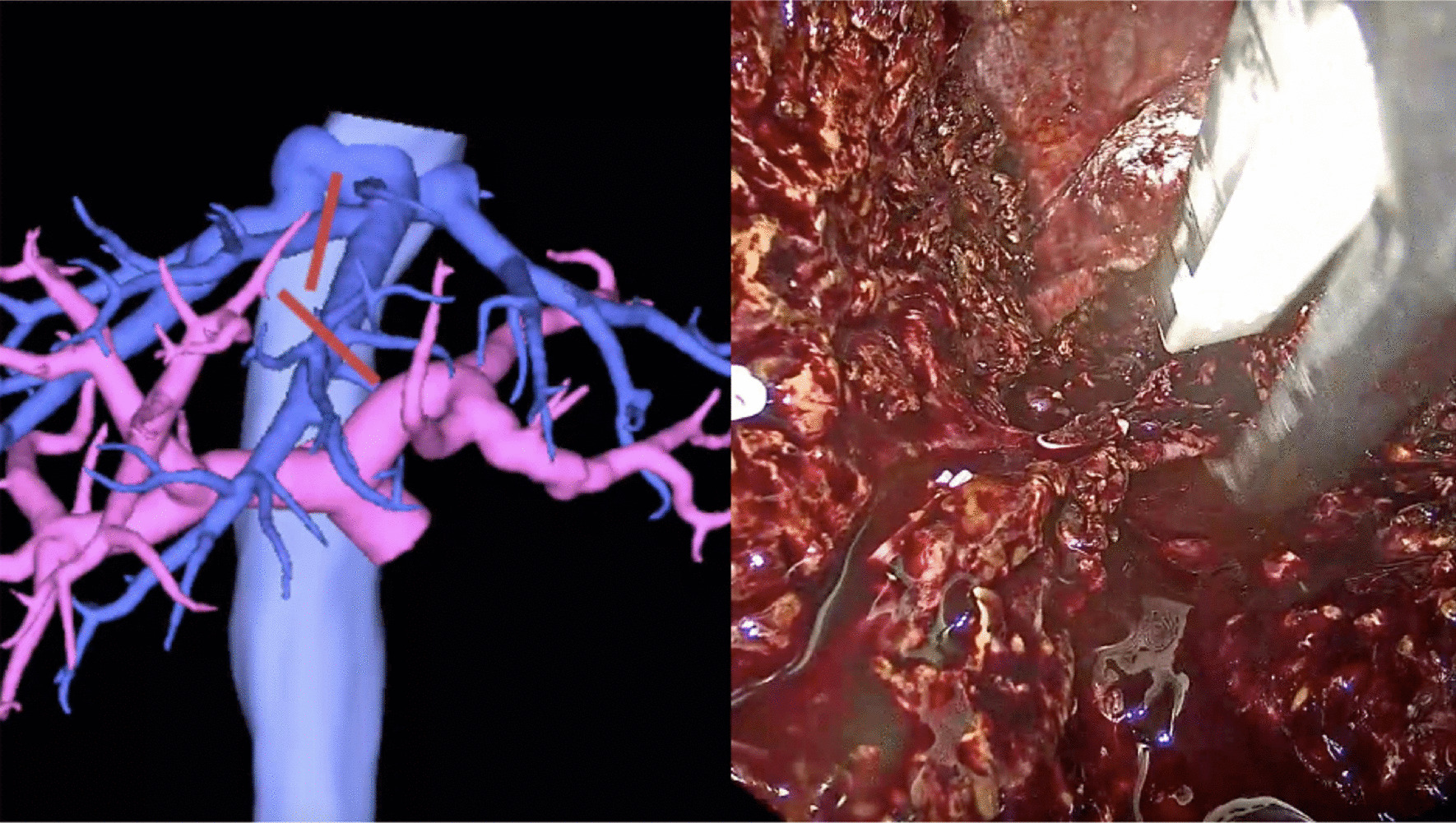
Middle hepatic vein after parenchymal transection and ligation for preoperative planning

**Fig. 4:**
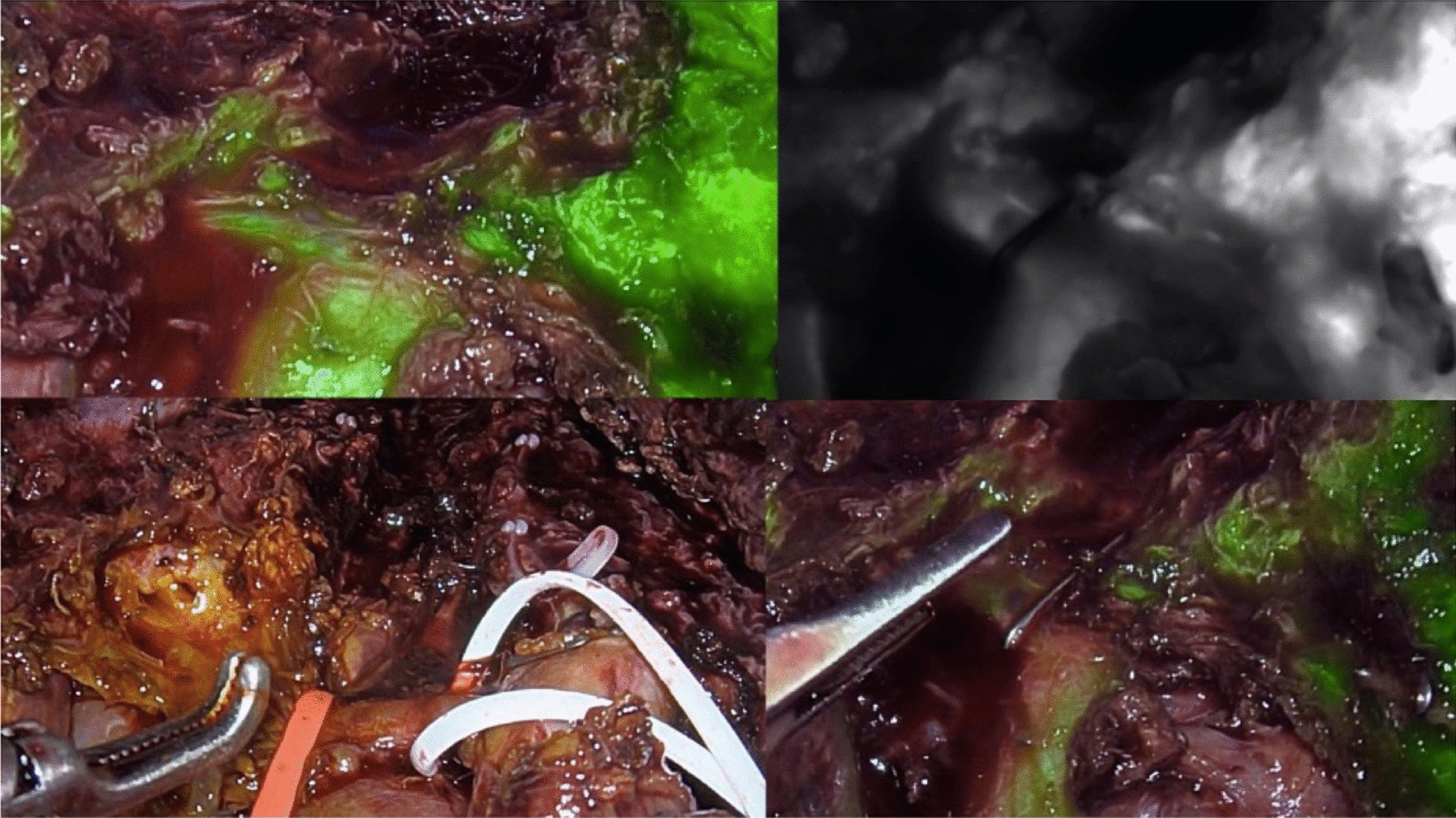
Real-time ICG cholangiogram-assisted bile duct division in pure laparoscopic donor right hepatectomy

Parenchymal transection was started from the superficial layer with an ultrasonic scalpel and then the deeper layers with a Cavitron ultrasonic surgical aspirator (CUSA Excel, Integra, United States). The Pringle maneuver was not routinely used. Parenchymal transection was performed following the middle hepatic vein (MHV). V5, V8 or the distal part of MHV was ligated as part of the preoperative planning (Fig. 3). Parenchymal transection was performed until the hilar plate and right intrahepatic duct were clearly identified. ICG fluorescence cholangiography was routinely used instead of conventional cholangiogram after 1 January 2020. ICG fluorescence cholangiography facilitated the real-time identification of the right intrahepatic duct (RHD) and bile duct confluence. The RHD was precisely double-clipped using 10 mm titanium clips and divided (Fig. 4). After that, the caudate lobe was split, and the remaining liver parenchyma was transected.

A Pfannenstiel incision was created and covered with a large wound protector and sealing cap. Pneumoperitoneum was re-established, and the right liver graft was covered with a plastic bag. Heparin was administered intravenously (70 units per kg), and following a 5-min wait, the RHA was double ligated with a titanium clip, the RPV was ligated with an endovascular staple, and the right hepatic vein (RHV) and inferior hepatic vein were also ligated with endovascular staples. Finally, the Makuuchi ligament was divided with an endovascular stapler. Then, the liver graft was removed via a plastic bag through a Pfannenstiel incision.

Finally, the heparinized effect was routinely reversed by intravenous protamine sulfate. The Pfannenstiel incision was closed layer by layer, and the pneumoperitoneum was re-established. Any bleeding was secured, and the left lobe of the liver was fixed to the abdominal wall using a suture. One 10 mm Jackson-Pratt drain was routinely placed in the right upper quadrant.

### Statistical analysis

The baseline characteristics and surgical outcomes of the donors and recipients between the CODRH and PLDRH groups of this study were compared. The distribution of normality was tested by the Kolmogorov–Smirnov test**.** Normally distributed continuous data are reported using the mean ± standard deviation and were compared using t-tests**.** Non-normally distributed continuous data are reported using the median ± interquartile range and were compared by the Mann–Whitney U test**.** Categorical data are reported as numbers (percentages) and were compared using Fisher’s exact test**.** For all analyses, a P-value of less than 0.05 was considered statistically significant**.** All statistical analyses were performed using SPSS version 23.0.0, IBM.

## Results

### Comparison of donor characteristics and outcomes between the CODRH and PLDRH groups

From 2015 to 2021, there were 56 cases of liver transplantation in the department. Thirty of those patients underwent adult LDLT and were operated on using a modified right lobe graft. There were 21 cases of CODRH and 9 cases of PLDRH. The baseline characteristics of the liver donors and donor complications are shown in Table 1. There was no significant difference between these two groups. Median graft volume in the PLDRH group was 693.8 ml. which was not significantly different from the CODRH group (726.5 ml). There were 8 of the donors in CODRH who had bile duct variation, which resulted 8 liver grafts in this group had multiple bile duct openings. In PLDRH group, There was one donor who had bile duct and portal vein variation. However, 3 liver grafts in this group had multiple bile duct orifices. The actual graft weight and GRWR were not significantly different between the groups. Overall complications after donor right hepatectomy were not significantly different between the two groups (33.3 vs 22.2%, p = 0.555). There were 2 (9.6%) donors in the CODRH group who experienced major complications. The most common complication in the CODRH group was transient biliary leakage which was spontaneously resolved. There were no complications in the CODRH group after donor13. However, there were two donors in the PLDRH group who had major complications (the 3rd and 7th donors of the PLDRH series). There were no grade IV or V complications in our series. The postoperative length of hospital stay was shorter in the PLDRH group than in the control group but had no statistical significant (8 vs 5 days, p = 0.115).

**Table 1:** Baseline characteristics and donor complications

Variables	CODRH (21)	PLDRH (9)	p-value
Baseline characteristics and donor complications			
Sex, male, n (%)	8 (38.1)	1 (25)	0.617
Relationship			0.063
Spouse	8 (38.1)	1 (11.1)	
Son or daughter	11 (52.4)	6 (66.7)	
Sibling	2 (9.5)	2 (22.2)	
Blood group			0.864
A	4 (19)	2 (22.2)	
B	4 (19)	1 (11.1)	
O	13 (62)	6 (66.7)	
AB	0	0	
Donor age	41 (31.5–48)	35.0 (28.5–42.0)	0.486
Donor BMI	21.7 (20.7–24.9)	22.6 (20.8–23.8)	0.615
Graft volume	726.5 (616.0–826.2)	693.8 (636.9–788.0)	0.980
% liver remaining	31.1 (29.1–35.4)	34.9 (31.9–37.2)	.118
Bile duct variation	8 (38.1)	1 (11.1)	0.815
Number of bile duct openings			0.804
Single	13 (61.9)	6 (66.7)	
Multiple	8 (38.1)	3 (33.3)	
Portal vein variation	3 (14.3)	1 (11.1)	0.593
Hepatic artery variation	3 (14.3)	2 (22.2)	0.593
Graft weight	667.0 (582.5–832.5)	593.0 (512.3–687.3)	0.244
GRWR	1.1 (1.0–1.4)	1.3 (0.9–1.5)	0.374
Postoperative outcomes and donor complications			
Overall complications, n (%)	7 (33.3)	2 (22.2)	0.555
C-D grade I	1 (4.8)	0	
Prolonged hyperbilirubinemia			
C-D grade II	4 (19.2)	0	
Bile leakage	3 (14.4)		
Chyme leakage	1 (4.8)		
C-D grade IIIa	1 (4.8)	1 (11.1)_	
Intra-abdominal collection	1 (4.8)	1 (11.1)_	
C-D grade IIIb			
Hepatic duct confluence injury	1 (4.8)	1 (11.1)	
Bleeding	1 (4.8)	1 (11.1)	
C-D grade IV to V	0	0	
Length of hospital stay, days	8.0 (7.0–9.0)	5.0 (5.0–7.0)	0.115

*BMI* body mass index, *GRWR* graft to recipient weight ratio, *C-D* Clavien-Dindo classification

Perioperative data and laboratory investigations of donors are shown in Table 2. The operative time in the PLDRH group was longer than that in the CODRH group but had no statistical significant (487.5 min vs 425.0 min, p = 0.197). Median blood loss was not significantly different between the two groups. There was a 19% rate of intraoperative packed red cell (PRC) transfusion in the CODRH group and 11.1% in the PLDRH group. There was no open conversion in the PLDRH group. Preoperative and postoperative laboratory investigation was not significantly different between the two groups.

**Table 2:** Perioperative data and laboratory parameters of donors

Perioperative data and laboratory investigation			
Variables	CODRH (21)	PLDRH (4)	p-value
Operative time	400 (317.5–510.0)	425.0 (395.0–487.5)	0.197
Blood loss	500 (350–700)	500.0 (300.0–7 50.0)	1.000
Packed red cell transfusion	4 (19)	1 (11.1)	0.593
Preoperative laboratory investigation			
Hemoglobin, g/dL	12.8 (11.8–14.8)	12.9 (12.5–13.8)	0.683
Total bilirubin, mg/dL	0.4 (0.3–0.5)	0.4 (0.3–0.8)	0.683
AST, IU/L	16.0 (14.5–18.5)	16.0 (13.5–21.0)	0.951
ALT, IU/L	15.0 (10.5–21.0)	12.0 (12.0–15.0)	0.615
Postoperative laboratory investigation			
Hemoglobin, g/dL			
Lowest Hb	10.9 (9.6–11.5)	10.7 (8.7–11.7)	0.867
Delta Hb (%)	− 16.9 (− 23.3 to − 10.7)	− 16.6 (− 35.4 to − 7.35)	0.234
Total bilirubin, mg/dL			
Peak Tb	2.3 (1.8–4.0)	1.6 (1.3–2.7)	0.115
Delta Tb (%)	5.2 (3.0–9.5)	3.5 (2.4–4.5)	0.208
AST, IU/L			
Peak AST	259.0 (187.0–347.0)	244.0 (172.5–272.0)	0.549
Delta AST (%)	13.4 (9.5–21.2)	12.3 (8.4–17.4)	0.486
ALT, IU/L			
Peak ALT	217.0 (154.0–375.0)	242.0 (172.0–275.5)	0.486
Delta ALT (%)	14.8 (7.7–23.6)	17.6 (9.3–21.0)	0.867

*AST* aspartate aminotransferase, *ALT* alanine aminotransferase

### Comparison of recipients’ characteristics and outcomes between the CODRH and PLDRH groups

#### Peri-operative

Perioperative data and outcomes of recipients are shown in Table 3. The preoperative data were not significantly different between the two groups. Half of the patients in each group were diagnosed with hepatocellular carcinoma. The median Model for End-Stage Liver Disease–Sodium (MELD-Na) score was slightly higher in the PLDRH group (9.5 vs 10, p = 0.152). However, the length of ICU and hospital stay were not significantly different (5 vs 7 days and 26 vs 24.5 days, p = 0.867 and 0.660, respectively). The incidence of major complications was not significantly different between the two groups (71.3 vs 55.6%, p = 0.792). The most common major complication in the CODRH group was biliary-related complications, such as biliary leakage or bile duct anastomotic stricture. There were two perioperative deaths in the CODRH group that resulted from acute hepatic artery thrombosis from aortic dissection and fatal ventricular arrythmia. There was one perioperative death in the PLDRH group that resulted from severe sepsis on postoperative day 13. Hemoculture revealed carbapenem-resistant *E. coli* septicemia. The detail of recipients who had mortality were showed in Table 4.

**Table 3:** Perioperative data and recipient complications

Perioperative data and recipient complications			
Variables	CODRH (21)	PLDRH (9)	p-value
Sex, male, n (%)	14 (66.7)	7 (77.8)	0.543
Age	59.0 (49.5–62.5)	57.0 (39.0–60.5)	0.749
Body weight	62.0 (57.5–71.0)	60.0 (45.0–76.0)	0.683
Etiology of cirrhosis, n, (%)			0.555
HBV	4 (19)	0	
HCV	7 (33.3)	5 (55.6)	
Alcohol	3 (14.3)	1 (11.1)	
Acute liver failure	1 (4.8)	1 (11.1)	
Other	6 (28.6)	2 (22.2)	
Hepatocellular carcinoma	11 (52.4)	5 (55.6)	0.873
MELD-Na score	9.5 (7.5–14.0)	10.0 (8.0–29.25)	0.152
ICU stay, days	5.0 (3.5–7.0)	7.0 (5.0–8.5)	0.867
Length of hospital stay, days	26.0 (19.0–65.5)	24.5 (15.7–30.3)	0.660
Major complications, n (%)	15 (71.3)	5 (55.6)	0.792
C-D grade IIIa	8 (38.1)	3 (33.3)	
C-D grade IIIb	2 (9.5)	1 (11.1)	
C-D grade IV	3 (14.2)	0	
C-D grade V	2 (9.5)	1 (11.1)	
30-day mortality	2 (9.5)	1 (11.1)	0.894
90-day mortality	5 (23.8)	1 (11.1)	0.426

*HBV* hepatitis B virus, *HCV* hepatitis C virus, *ICU* intensive care unit, *MELD-Na* Model for End-Stage Liver Disease–Sodium score, *C–D* Clavien–Dindo classification

**Table 4:** The detail of recipients who had mortality

Recipient number	Type of donor hepatectomy	Age	Meld-NA	Posttransplant survival (days)	Cause of death
3	CODRH	59	19	69	Aortic dissection
9	CODRH	62	9	90	Sepsis
17	CODRH	62	8	20	Sepsis
18	CODRH	53	9	18	Hepatic artery thrombosis
20	CODRH	48	35	3	Cardiac arrythmia
25	PLDRH	48	11	13	Sepsis

## Discussion

The aim of this article is to report the initial experience and outcomes of adult LDLT in a low-volume liver transplant center in a developing country and propose a strategy to develop PLDRH in these circumstances. The donor characteristics, perioperative data, donor outcomes and recipient outcomes were compared between CODRH and PLDRH.

The baseline characteristics of donors were not different between the 2 groups. The median graft volume in the PLDRH group was 693.8 mL. which is not different from the CODRH group. Previously, there were donor selection criteria proposed by Kim et al. [8] in which graft weight should be smaller than 500 g due to the difficulty of graft manipulation and the largest parenchymal transection areas. However, our result corresponded to a study from Lapisatepun et al. [9], which showed that graft weight affects neither donor outcomes nor graft function after PLDRH. The elevation of aspartate aminotransferase (AST) postoperatively may be caused by hepatocyte injury from liver mobilization and parenchymal transection [10, 11]. Some reports have shown that PLDRH causes a higher elevation of AST than CODRH [12]. Nevertheless, peak AST and delta AST in this study were not significantly different between the groups. We believe that a large graft size should not be a contraindication for performing PLDRH but may increase the difficulty of the procedure, especially for surgeons.

There was no bile duct variation from preoperative MRCP to multiple bile duct opening in the CODRH group. However, there were 3 patients in the PLDRH group who had multiple bile duct openings to reconstruct, while all donors had a single short right hepatic duct (< 5 mm) from preoperative MRCP. This may have resulted from the bile duct division and closure technique in donor operation. Our technique used a double 10 mm titanium clip to close the right hepatic duct stump in the PLDRH group, causing an inevitable loss of approximately 3–4 mm in length, in contrast to the CODRH group, which used a polydioxanone suture (PDS) 6/0 closure and resulted in a loss of only 1–2 mm. of length. Our result correlated with a study from a high-volume PLDRH center, which also showed an increased frequency of multiple bile duct openings in the PLDRH group [4]. Biliary ductoplasty should be considered a valuable option to overcome biliary complications in the recipient.

The operation time in the PLDRH group was longer than that in the CODRH group. Many studies have reported the same result [13–15]. The estimated blood loss in the PLDRH group was not significantly different from that in the CODRH group. Many studies show the benefit of pneumoperitoneum, which helps reduce potential blood loss in laparoscopic liver resection as well as laparoscopic donor hepatectomy [16]. PRC transfusion was used in donors 2–4 and 17 in the CODRH group with estimated blood loss of more than 1000 mL, which can occur during the starter phase of the LDLT program. However, the lowest hemoglobin and delta hemoglobin levels were not significantly different between the two groups.

Regarding postoperative outcomes and donor complications, there were 2 donors in the CODRH group who experienced major complications. Donor number 3 required percutaneous drainage due to intra-abdominal collection, and donor number 7 required immediate biliary reconstruction due to a hepatic duct confluence injury during bile duct transection. There were no major or minor complications in the CODRH group after donor 12. The team started using an ICG cholangiogram in CODRH instead of conventional cholangiograms in donor number 21. After that, we started using ICG cholangiograms to facilitate real-time bile duct division in PLDRH. There were 2 donors in the PLDRH group who had major complications: intra-abdominal collection required percutaneous drainage, and postoperative bleeding required repeat laparoscopy to stop the bleeding. Fortunately, all donors recovered well, and there were no consequent complications during the long-term follow-up period. We believe that the ICG cholangiogram is a gamechanger that facilitates the smooth transition from CODRH to PLDRH and obviously beneficial to the donor. In our study, there were no biliary complications among donors in the PLDRH cohort. However, there were four donors (14.4%) in the CODRH cohort who had biliary complications, and one of them (4.8%) had an injury affecting the confluence of the bile ducts, which was corrected with Roux-en-Y hepaticojejunostomy. Our hypothesis is that, while using a real-time ICG cholangiogram, the surgeon can simultaneously perform bile duct dissection, which will result in precise division of the bile ducts. For conventional cholangiography, the fluoroscope needs to move in and out of the operative field, which may cause some error during bile duct division. We strongly believe that dynamic ICG cholangiography is a transformative technology for donor surgery using both laparoscopic and open approaches.

As the small volume liver transplant center, we try our best to build the safe and sustainable LDLT program. Back to 2010, Our hospital was first started our adult LDLT and five cases were performed but unfortunately the mortality rate was high as 60% which resulted from early allograft dysfunction, vasculo-biliary complication or even chronic allograft rejection. The leader transplant surgeon at that time who was not HB surgeon was then departed which resulted in the shutting down of transplant program. The many abdominal transplant programs might lead by transplant surgeon who has proficiency in liver, pancreas and kidney transplantation. The vascular surgeon can also lead the transplant program due to familiarity with the vascular technique. However, the adult LDLT is one of overlapping area of transplant and HB surgery. The comprehensive knowledge which includes liver biology, anatomy and complex hepatectomy skill are necessary for building the sustainable and successful LDLT team. We decided to send the young HB surgeon (SJ) who had the extensive experience in complex hepatobiliary surgery which included major hepatectomy and vascular reconstruction in cholangiocarcinoma surgery to United states and South Korea to obtain the experience in both deceased donor and living donor liver transplantation. After he came back, the first DDLT was performed in November 2014 and after the successful of 3 cases of DDLT, the first adult LDLT was performed in July 2015. Afterwards, the LDLT is a majority of transplant volume in our center (Additional file 1) which was not only keep the competency of all transplant team but also decrease the gap between demand and supply of deceased organ donation in the low organ donation rate country.

Additionally, For surgical procedures, the case volume is strongly associated with the outcome. Outcome of ALDLT which is well recognized to be one of the most complex is also improved after 15–20 cases [17]. The impact of case volume is not just only to surgical skill of surgeon but also effect to patient-donor selection and post-operative management which relate to patient and graft survival. One of the advantage of LDLT is the program can maintain case volume which is added on to volume from DDLT. In our center, the 90 days mortality has gradually decreased from 20–30% in the first 20 cases to 0% in the last 10 cases. (Additional file 2) We would like to encourage the small liver transplant program to start ALDLT and keep maintaining adequate case volume which will subsequently improve the patient’s outcome.

Currently, our center is the only active adult living donor liver transplantation in Thailand, but the transplant volume is still low, performing 10–12 cases of adult liver transplantation annually. Our center also started minimally invasive liver surgery in 2015 and gradually developed surgical techniques for major laparoscopic liver resection. PLDRH was started in 2020 after the main donor surgeon (WL1), who had extensive experience in both open and laparoscopic complex hepatobiliary surgery and CODRH received adequate training from a high-volume minimally invasive liver transplantation center that used the reproducible technique [18–21]. Unlike several previous studies [22, 23], our center did not start PLDRH with hand- or laparoscopic-assisted donor hepatectomy procedures. We strongly believe that PLDRH can be initiated upfront if these strategies are implemented.

**Fig. 5:**
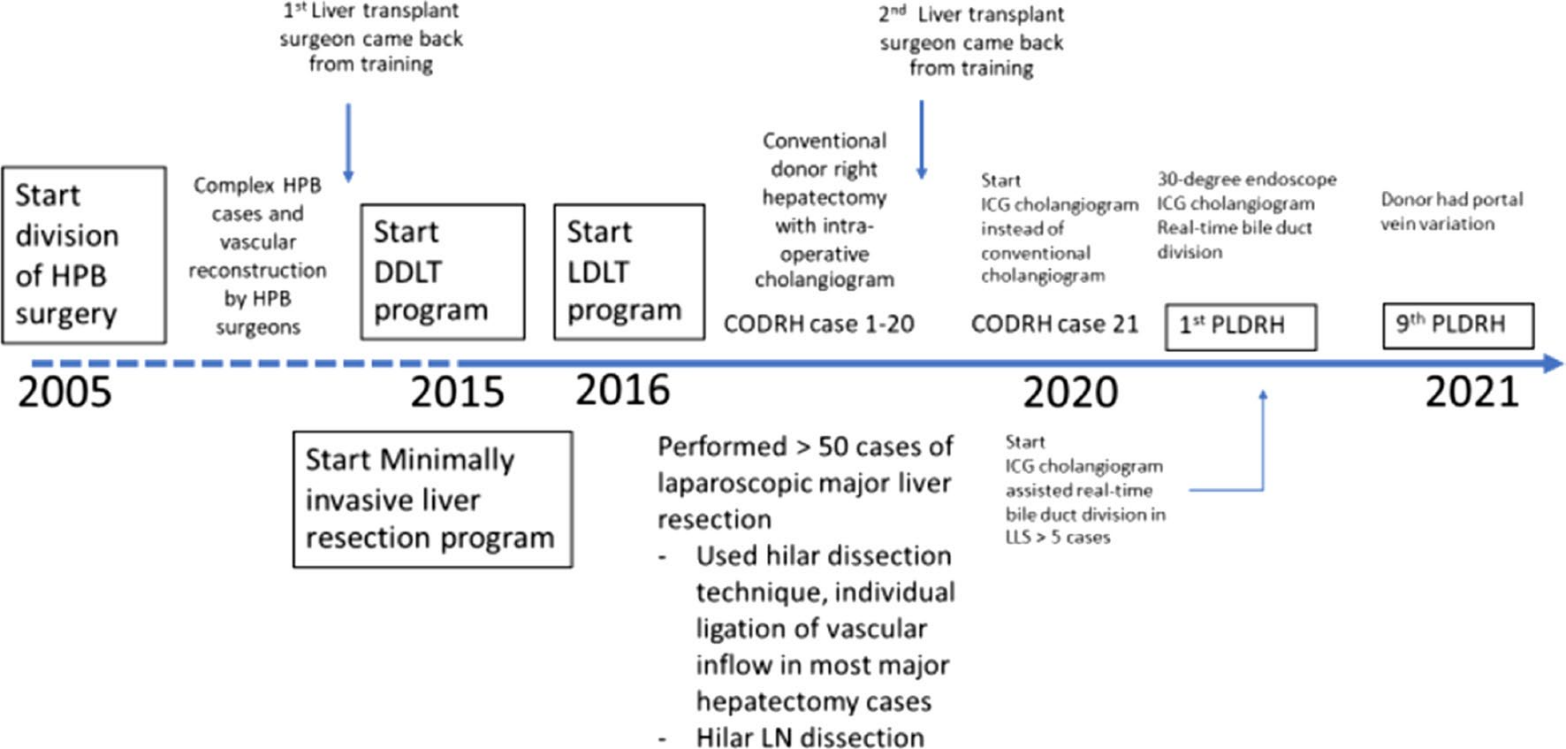
Summary of strategy to develop PLDRH in low-volume transplant centers

The strategy to convert from CODRH to PLDRH in small liver transplant centers in developing countries has several steps, which are summarized in Fig. 5. First, as a high-volume hepatobiliary and minimally invasive surgery center, the institution has a surgical team that has already mastered the laparoscopic skill required for PLDRH, especially hilar dissection and hilar structure identification. Second, there is the implementation of the skills required for PLDRH from laparoscopic hepatectomy for cancer cases. Examples include familiarity of using a near-infrared fluorescence camera for ICG cholangiograms to assist in bile duct division in cancer cases or parenchymal transection skills in laparoscopic major hepatectomy. These techniques are very useful for PLDRH. Moreover, extensive training in a high-volume center that has a reproducible PLDRH technique is mandatory for donor surgeons who already have experience in CODRH.

The type of laparoscope is also very important for PLDRH starters. Many high-volume centers favor a 3-dimensional (3D) flexible endoscope over a rigid endoscope due to the flexibility of the camera angle and depth of perception [19]. However, our team solely uses a 30-degree, 2-dimensional (2D) ICG endoscope for PLDRH for several reasons. First, the endoscope operator in this department is the rotational surgical resident who is not familiar with flexible 3-scope manipulation. Second, a 30 degree 2-D ICG endoscope can be used as a single camera system for the entire operation. Last, familiarity with the 2D images could overcome the lack of depth perception by the 3D imaging system.

Many studies have shown that PLDRH has several advantages over CODRH, such as reduced donor recovery times, better cosmetic results, and improved quality of life without compromising the quality of the liver graft and donor safety [4, 5, 16]. However, these studies are usually reported from a few high-volume LDLT centers, which probably made the low-volume transplant center reluctant to start PLDRH. This study reported the feasibility of PLDRH in low-volume transplant centers, which was comparable to CODRH. To the best of our knowledge, this is the first PLDRH report from Southeast Asia.

The major complications and 30-day and 90-day mortality of recipients in his study were relatively high compared to the results from high-volume centers. A majority of the patients died from infection, which may be related to surgical technique. Four recipients in the CODRH group who underwent surgery during the initial phase of the program died, two of them from unpreventable causes (aortic dissection and cardiac arrythmia). The learning curve of the team, which included patient selection, surgical technique, and perioperative care, was the main influence on the short- and long-term outcomes of transplant patients. We believe that posttransplant survival will improved when the program is able to maintain a median transplant volume (more than 10 cases/year) [24],which will lead to improved patient and donor selection as well as an increase in team competency, which would apply not only to the surgeon but also to the hepatologist, endoscopist, interventional radiologist and nurses. There was a large volume single center study showed that PLDRH group had significantly higher incidence of long-term biliary complication^4^. So, we could not confidentially conclude that the complication in both groups were not significantly difference due to shorter follow up time and lower volume of LDLT. Several studies mention that the number of cases of LDLT affects the result of the transplantation program [25–27]. We strongly believe that morbidity and mortality will be lowered if the annual transplant volume is increased.

This study had several limitations. First, this report had a small number of cases in both groups; thus, the sample size was not sufficient to obtain a statistically significant result. Second, there is a potential bias in the study due to the retrospective nature of this study and the selection bias in the selection criteria. Finally, the long-term outcomes of donors and recipients of PLDRH groups were not examined in this study, despite their critical importance.

## Conclusion

The results of our initial experience with PLDRH showed that PLDRH is safe and beneficial for donors without compromising graft function. PLDRH can be attempted in centers with high levels of experience in minimally invasive hepatobiliary surgery and well-established adult LDLT programs. However, starting a PLDRH program without changing from a hand-assisted or laparoscopic-assisted program has limited evidence and should be done cautiously.

## Supplementary Information

**Additional file 1. **The total liver transplant volume from 2009 to 2021.

**Additional file 2. **The case number and 90 day mortality.

## Data Availability

The datasets generated during and analysed during the current study are not publicly available due to the rules and regulations of our hospital but are avail- able from the corresponding author on reasonable request.

## Abbreviations

AST: Aspartate aminotransferase
ALT: Alanine aminotransferase
CHD: Common hepatic duct
CODRH: Conventional open donor right hepatectomy
GRWR: Graft to recipient weight ratio
ICG: Indocyanine green
IVC: Inferior vena cava
LDLT: Living donor liver transplantation
LLS: Left lateral sectionectomy
MELD-Na: Model for End-Stage Liver Disease–Sodium
MHV: Middle hepatic vein
OLR: Open liver resection
PDS: Polydioxanone suture
PLDRH: Pure laparoscopic donor right hepatectomy
PLDRH: Pure laparoscopic donor right hepatectomy
RHA: Right hepatic artery
RHD: Right intrahepatic duct
RHV: Right hepatic vein
RPV: Right portal vein
2D: 2-Dimensional
3D: 3-Dimensional
