# Supplementary figures and images for: Comparative outcomes of pure laparoscopic and open donor right hepatectomy: the first report from a Southeast Asian transplant center
Source: BMC Surg. 2022 Feb 11;22:48. doi: 10.1186/s12893-022-01507-0 (PMC8832827; doi:10.1186/s12893-022-01507-0)

Supplementary data1: The total liver transplant volume from 2009 – 2021

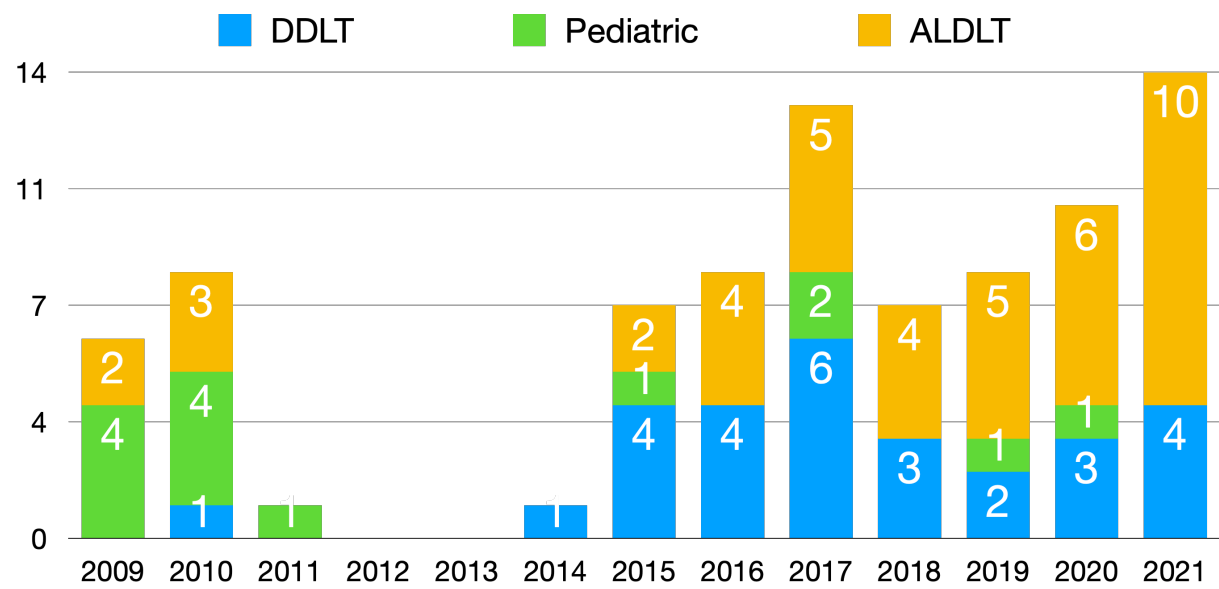

Supplement: Supplementary file 1 — Additional file 1. The total liver transplant volume from 2009 to 2021. [file 12893_2022_1507_MOESM1_ESM.pdf]
