# Supplementary material for: Comparative outcomes of pure laparoscopic and open donor right hepatectomy: the first report from a Southeast Asian transplant center
Source: BMC Surg. 2022 Feb 11;22:48. doi: 10.1186/s12893-022-01507-0 (PMC8832827; doi:10.1186/s12893-022-01507-0)
Supplement: Supplementary file 2 — Additional file 2. The case number and 90 day mortality. [file 12893_2022_1507_MOESM2_ESM.pdf]

Supplementary data 2: The case number and 90 day mortality

Case numbers	*Year*	*90-day mortality*
1-10	2015-2017	20%
11-20	2017-2019	30%
21-30	2019-2021	10%
31-37	2021-now	0%
